# The correlation between temperature and the incidence of acute ischaemic stroke in Yanji, China: a time series study

**DOI:** 10.3389/fpubh.2025.1568759

**Published:** 2025-07-29

**Authors:** Binyu Zhao, Yao Zhao, Shuang Wang, Meng Tan, Jianguo Pei, Ruojin Li

**Affiliations:** ^1^Department of Biostatistics, School of Public Health, Harbin Medical University, Harbin, China; ^2^Department of Radiology, Affiliated Hospital of Yanbian University, Yanji, China

**Keywords:** acute ischaemic stroke, distributed lag nonlinear model, temperature, minimum morbidity temperature, meteorological conditions

## Abstract

**Objective:**

Acute ischaemic stroke (AIS) is a common cerebrovascular disease; however, the relationship between temperature and its onset remains unclear. Therefore, we aimed to explore the association between temperature and the incidence of AIS in Yanji, a city in Northeast China.

**Methods:**

We collected data on patients with AIS from the Affiliated Hospital of Yanbian University from Jan 1, 2019, to Dec 31, 2023, along with meteorological data during the same period. A distributed lag nonlinear model (DLNM) was constructed to estimate the correlation between temperature and the risk of developing AIS, and further subgroup analyses stratified by sex, age and period (non-pandemic, pandemic) were performed.

**Results:**

A total of 15,997 patients were diagnosed with AIS during the study period. Using the minimum morbidity temperature (MMT) of −8.3°C as a reference, extreme heat (26.5°C, 99th percentile of temperature) and moderate heat (21.9°C, 90th percentile of temperature) were found to increase the risk of developing AIS on the day of exposure, with this effect persisting even after a lag of 7 days. The relative risks (RRs) with 95% confidence intervals were 1.268 (95% CI: 1.143–1.407) and 1.239 (95% CI: 1.145–1.341), respectively. In contrast, no harmful effects were observed within a lag of 0–7 days for extreme cold (−17.4°C, 1st percentile of temperature) and moderate cold (−10.6°C, 10th percentile of temperature) conditions. Subgroup analysis revealed that in the early stages of exposure to extreme heat and moderate heat, the risk of developing AIS increased in males and individuals under 65 years of age, and there were differences in the risk of developing AIS between the pandemic and non-pandemic periods.

**Conclusion:**

Our research suggests that exposure to high-temperature environments increases the risk of developing AIS and that the harmful effects of high temperatures have a lag effect. Reducing exposure to high temperatures may help alleviate the medical burden associated with AIS.

## 1 Introduction

Stroke is a prevalent cerebrovascular disease in clinical practice characterized by rapid onset, a high mortality rate, and poor prognosis and poses a major challenge to global public health (1, 2). According to the Global Burden of Disease (GBD) Study 2021, stroke is the third leading cause of death worldwide (3). In China, it has become the leading cause of death and disability (4, 5). According to the China Stroke High-risk Population Screening and Intervention Program 2020, there were 3.4 million new cases of stroke, 2.3 million deaths, and 17.8 million patients in China, with a standardized prevalence rate of 2.61%. The prevalence rates of ischaemic and haemorrhagic stroke were 2.27 and 0.39% (6, 7), respectively. The disease is associated with a poor prognosis and is often accompanied by speech (8), motor (9), and visual impairments (10), which can easily lead to swallowing difficulties (11), joint contractures (12), and significant declines in both self-care ability and quality of life. Additionally, emotional issues, including depression and anxiety (13), may also occur.

Previous studies have revealed a significant association between temperature and stroke incidence or mortality. The relationship between temperature and stroke is nonlinear and usually manifests as a V-, U-, or J-shaped curve (14–16), suggesting that both extremely low and high temperatures can increase the risk of stroke incidence or mortality. In Central Europe, decreasing temperatures have been associated with an increased incidence of stroke; this trend is particularly evident among older adults (17). Similarly, in Canada, low temperatures have also been confirmed to be closely associated with the occurrence of stroke, especially among males (18). Additionally, several studies have pointed out that high temperatures are also an important risk factor for stroke (16, 19, 20). In extremely hot environments, the risk of stroke can rise rapidly within a few hours, and its adverse effects may last for several days. Notably, the impact of temperature may differ between stroke subtypes, such as ischaemic and haemorrhagic stroke.

Ischaemic stroke is the most prevalent type of stroke, accounting for 60–80% of all cases (21–23). As the largest developing country in the world, China is facing major public health challenges brought about by climate change, including the rising incidence and increasing medical burden of ischaemic stroke (7). However, recent research on the relationship between temperature and ischaemic stroke has primarily focused on the warmer southern regions of China. Studies examining the association between urban temperature and acute ischaemic stroke (AIS) in Northeast China remain limited. Given the region’s high latitude and pronounced seasonal temperature variations, the region offers unique conditions for studying the association over a broader temperature range. Therefore, the present study aimed to investigate the relationship between ambient temperature and AIS incidence in Yanji, a city located in Northeast China. Specifically, we employed a distributed lag nonlinear model (DLNM) to assess the cumulative lag effects of temperature on AIS and to estimate the overall impact of extreme temperatures. The findings will contribute to understanding the temperature–stroke relationship in a high-latitude urban setting, and provide scientific evidence for developing public health strategies, optimizing healthcare resource allocation, and mitigating the impacts of extreme weather under climate change.

## 2 Materials and methods

### 2.1 Ethical statement

This study complies with the ethical guidelines of the Affiliated Hospital of Yanbian University and was approved by the institutional ethics committee (Approval Number: No. 2025028). As the data used are aggregated and no individual participants were contacted, additional informed consent was not required.

### 2.2 Research area

Yanji (42°50 N to 43°23 N, 129°01E to 129°48E) is located in eastern Jilin Province, Northeast China, adjacent to the northern foothills of the Changbai Mountains (Supplementary Figure S1). It has four distinct seasons and a typical continental climate. The annual average temperature is 5.5°C, the annual average precipitation is 479.0 mm, the average sunshine duration is 2447.2 h, and 164 frost days occur each year (http://www.yanji.gov.cn/sq2473/dldm/).

### 2.3 Data collection

In the present study, we collected data from patients diagnosed with AIS at the Affiliated Hospital of Yanbian University from Jan 1, 2019, to Dec 31, 2023. The patient data were obtained from the hospital’s medical records and AIS was identified using the 10th revision of International Classification of Diseases (ICD-10) codes (I63.0-I63.9). During the preprocessing stage, patient names were anonymized to protect personal privacy. The data were organized to obtain the daily number of AIS cases, along with the sex and age information of the patients. Daily meteorological data (*N* = 1826, no missing) for the same period were obtained from the China Meteorological Administration (https://data.cma.cn/), including temperature (°C), relative humidity (%), precipitation (mm), wind speed (kph), and air pressure (hPa).

### 2.4 Statistical analysis

The distributed lag nonlinear model (DLNM) is a widely-used statistical modeling framework in time-series analysis (24). It was specifically designed to explore the complex relationships between exposure factors (such as environmental and meteorological factors) and health outcomes (such as disease incidence and mortality). The unique strength of the DLNM lies in its ability to simultaneously account for both the nonlinear exposure-response relationships and the lagged effects of exposure. This comprehensive consideration endows the model with significant application value across various fields, including environmental epidemiology, meteorology, and ecology. Previous studies have shown that the effect of temperature on the incidence of AIS is nonlinear and has a lag effect. Considering that the daily incidence of AIS in the general population is a low probability event that approximately follows a Poisson distribution (25), we used a quasi-Poisson generalized additive model (GAM) with a DLNM term to evaluate the effect of temperature on AIS occurrence. The model is as follows:


$$Y_{t}\sim \text{Poisson}(\mu_{t})$$

$$\log[E(Y_{t})]=\alpha +\beta T_{t,l}+\sum \mathrm{ns}(\text{factor},\mathrm{df})+\mathrm{ns}(\text{time},\mathrm{df})+\mathrm{DOW}$$

In the formula, *Y_t_* is the number of AIS cases on day *t*, *E*(*Y_t_*) is its expected value, *α* is the intercept, and *β* is the regression *T_t,l_* coefficient, which represents the temperature cross basis function. Considering the lag effect, the maximum number of lag days was set to 7, *ns* represents the natural spline curve, *df* is the degree of freedom, and the meteorological factors included in the study include relative humidity, precipitation, wind speed, and air pressure. The *df* is set to 3, and the *df* for time is 7 to control for long-term trends and seasonality. *DOW,* the indicator variable for the day of the week, was included in the model as a categorical variable.

We calculated the Spearman rank correlation coefficients between meteorological variables (Supplementary Table S1) and the variance inflation factor (VIF) values (Supplementary Table S2) to assess the risk of multicollinearity. VIF values < 5 indicated a lack of significant multicollinearity among the independent variables. Additionally, we performed model residual diagnostics to evaluate the model fit (Supplementary Figure S2). If the *p*-value of the residual test was > 0.05, then there was no sufficient evidence of autocorrelation among the residuals, and the model fit was considered to be valid. Based on these analyses, we concluded that there was no multicollinearity among the variables and that the model had a good overall fit.

We consulted previous studies and used the minimum morbidity temperature (MMT)—defined as the temperature associated with the lowest risk of AIS onset, determined as the minimum point of the overall cumulative exposure–response curve (Supplementary Figure S3)—as the reference temperature (25, 26, 27). This approach was chosen to address the wide temperature range (−22.6°C to 29.4°C) observed in the study area, where using the mean or median temperature as a reference might lead to over- or underestimation of the actual risk. In addition, we estimated the relative risk (RR) and 95% confidence interval (CI) of AIS onset with a lag of 0–7 days by comparing the risk at four specific temperatures—extreme cold (−17.4°C, 1st percentile), moderate cold (−10.6°C, 10th percentile), moderate heat (21.9°C, 90th percentile), and extreme heat (26.5°C, 99th percentile)—to that at the MMT.

One patient with a missing age in 2019 was excluded in the subgroup analysis. The analysis was performed using R software (version 4.1.2, R Development Core Team, Vienna, Austria) with packages including “dlnm,” “splines,” and “ggplot2.” All CIs are 95% CIs, and a two-sided *p* < 0.05 was considered to indicate statistical significance.

## 3 Results

### 3.1 Demographic characteristics and incidence trends

From Jan 1, 2019, to Dec 31, 2023, a total of 15,997 AIS cases were diagnosed at the Affiliated Hospital of Yanbian University. The annual number of cases was 2,173, 2,632, 3,203, 3,713, and 4,276, respectively, showing a significant increasing trend. The highest number of cases in a single day was recorded on Aug 1, 2023, with 27 cases. The majority of patients were male (*N* = 10,642, 66.52%), with ages mostly concentrated between 65–74 years (*N* = 5,420, 33.88%) and 55–64 years (*N* = 4,455, 27.85%) (Table 1).

**Table 1:** Demographic characteristics of AIS patients from 2019 to 2023.

Variable	2019	2020	2021	2022	2023
AIS Cases	2,173	2,632	3,203	3,713	4,276
Sex					
Male	1,464 (67.37%)	1770 (67.25%)	2,133 (66.59%)	2,492 (67.12%)	2,783 (65.08%)
Female	709 (32.63%)	862 (32.75%)	1,070 (33.41%)	1,221 (32.88%)	1,493 (34.92%)
Male/Female	2.06	2.05	1.99	2.04	1.86
Age^a^					
<35	14 (0.64%)	14 (0.53%)	30 (0.94%)	34 (0.92%)	36 (0.84%)
35–44	63 (2.9%)	79 (3%)	101 (3.15%)	93 (2.5%)	104 (2.43%)
45–54	318 (14.63%)	367 (13.94%)	378 (11.8%)	402 (10.83%)	432 (10.1%)
55–64	641 (29.5%)	752 (28.57%)	915 (28.57%)	1,037 (27.93%)	1,110 (25.96%)
65–74	659 (30.33%)	904 (34.35%)	1,074 (33.53%)	1,238 (33.34%)	1,545 (36.13%)
75–84	410 (18.87%)	442 (16.79%)	582 (18.17%)	777 (20.93%)	867 (20.28%)
85–94	65 (2.99%)	73 (2.77%)	118 (3.68%)	129 (3.47%)	174 (4.07%)
≥95	2 (0.09%)	1 (0.04%)	5 (0.16%)	3 (0.08%)	8 (0.19%)

^a^One patient whose age was missing in 2019.

Supplementary Figure S4 shows the monthly trend of AIS incidence during the study period. The data show that the number of AIS cases has increased from May to August each year, and the number of AIS cases in 2022 and 2023 has significantly increased compared with that in previous years.

### 3.2 Descriptive statistics of AIS case numbers and meteorological data

Supplementary Figure S5 presents time series plots of the meteorological variables during the study period. A statistical description of the number of AIS cases and meteorological factors is presented in Table 2. During the study period, there was an average of 8.8 cases of AIS per day, with the largest being 27 cases. The average temperature was 6.9°C, the lowest temperature was −22.6°C, the highest temperature was 29.4°C, the average relative humidity was 65.1%, the average precipitation was 0.2 mm, the average wind speed was 20.8 kph, and the average air pressure was 1015.7 hPa.

**Table 2:** Statistical description of AIS cases and meteorological factors during the study period.

Variable	Mean ± SD	Min	Percentage							Max
			1st	10th	25th	50th	75th	90th	99th	
AIS cases	8.8 ± 4.1	0	1	4	6	8	11	14	20	27
Meteorology										
Temperature, °C	6.9 ± 12.3	−22.6	−17.4	−10.6	−3.8	8.5	17.8	21.9	26.5	29.4
Relative humidity, %	65.1 ± 17.8	16.5	28.9	40.8	50.9	66.0	79.6	88.0	97.0	99.2
Precipitation, mm	0.2 ± 0.6	0	0	0	0	0	0.1	0.4	2.9	9.1
Wind speed, kph	20.8 ± 9.1	3.6	5.9	10.1	14.4	19.8	27.6	32.4	43.2	57.6
Air pressure, hPa	1015.7 ± 8.8	990.4	997.5	1004.3	1008.8	1015.3	1022.7	1027.3	1034.6	1037.2

### 3.3 Relationship between temperature and AIS incidence

We first determined the MMT, which has the least impact on the risk of developing AIS, and used it as a reference temperature (MMT = −8.3°C, RR = 1) to calculate the RR and its 95% CI of AIS onset at other specific temperatures.

**Figure 1:**
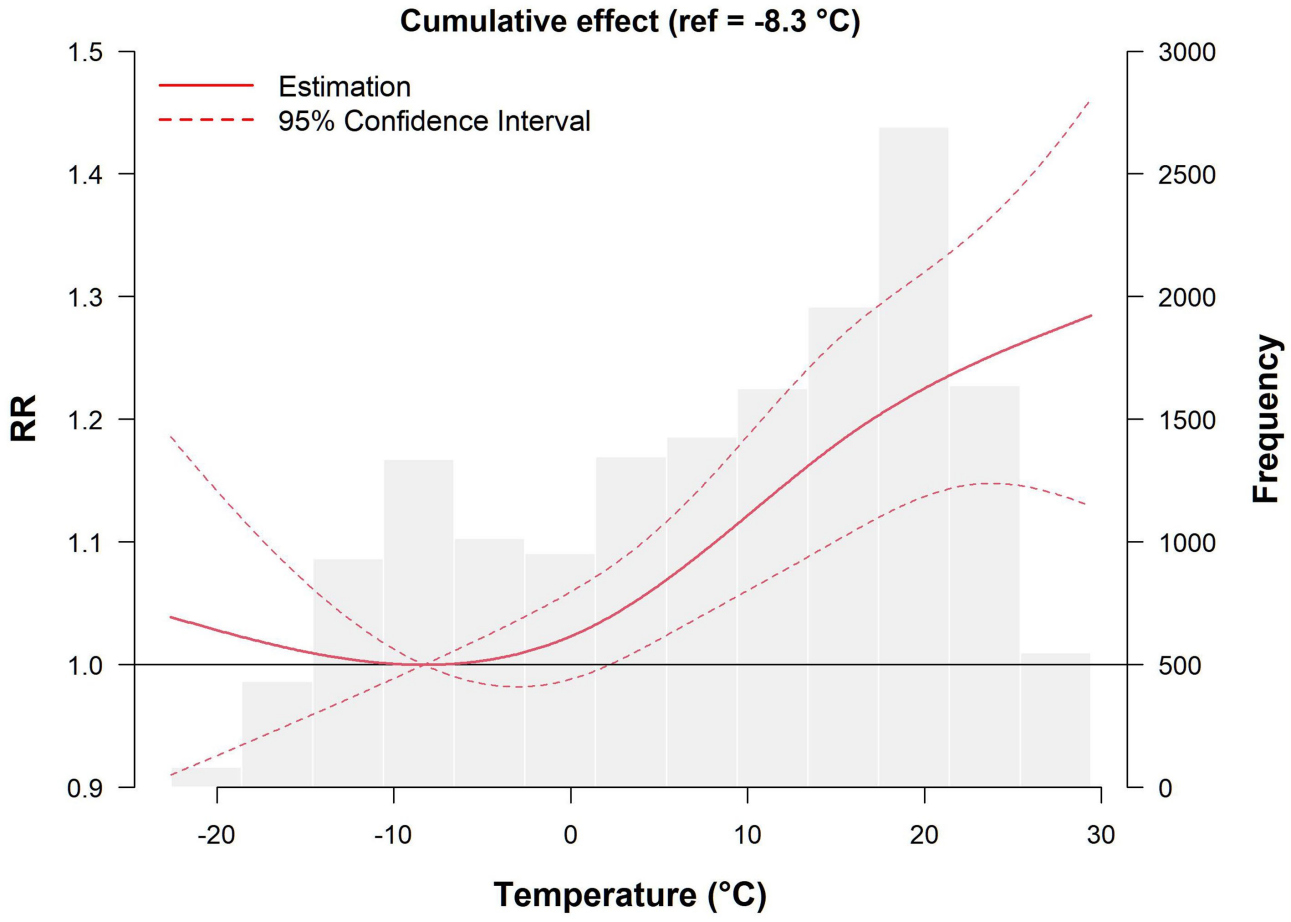
Cumulative effect of temperature on AIS incidence with a lag of 0–7 days.

The effect of temperature on the risk of developing AIS with a lag of 0–7 days is shown in Figure 1. Overall, the exposure–lag–response relationship between temperature and daily AIS incidence followed a nonlinear curve, and the histogram in the figure reflects the distribution of AIS cases at different temperatures. Compared with MMT, both low and high temperatures increased the risk of developing AIS, but the effect of high temperature was more pronounced. As the temperature gradually increased, the number of cases increased. When the temperature ranged from 17.4 to 21.4°C, the number of cases reached 2,692, and the risk of onset peaked at 29.4°C, with an RR of 1.284 (95% CI: 1.129–1.461).

The overall cumulative effects of extreme cold (−17.4°C), moderate cold (−10.6°C), moderate heat (21.9°C), and extreme heat (26.5°C) on the incidence of AIS after different numbers of lag days are shown in Table 3. Specifically, within a lag of 0–7 days, neither extreme cold nor moderate cold had statistically significant harmful effects, whereas the harmful effects of moderate heat on AIS onset were evident on the day of exposure and continued until lag0–3 days, with RR values of 1.139 (95% CI: 1.019–1.274) and 1.220 (95% CI: 1.032–1.443), respectively. Even when the number of lag days was extended to 7, harmful effects were still observed, with an RR of 1.239 (95% CI: 1.145–1.341). The pattern of extreme heat closely mirrors that of moderate heat, with RR values of 1.182 (95% CI: 1.040–1.343) on the day of exposure and 1.268 (95% CI: 1.143–1.407) within a lag of 0–7 days. The overall trend of moderate and extreme heat indicates that high-temperature exposure has an acute effect on the onset of AIS, and the harmful effects often reach their maximum in the lag phase (a lag of 0–2 days), accompanied by longer lag effects.

**Table 3:** Cumulative relative risks (RRs) of AIS incidence at specific temperatures.

Lag	Extreme cold −17.4°C, 1st	Moderate cold -10.6°C, 10th	Moderate heat 21.9°C, 90th	Extreme heat 26.5°C, 99th
Lag0–0	0.970 (0.911, 1.032)	0.992 (0.979, 1.006)	1.139 (1.019, 1.274)	1.182 (1.040, 1.343)
Lag0–1	0.960 (0.877, 1.050)	0.990 (0.970, 1.009)	1.221 (1.038, 1.438)	1.289 (1.070, 1.552)
Lag0–2	0.966 (0.880, 1.061)	0.991 (0.970, 1.011)	1.243 (1.047, 1.476)	1.311 (1.079, 1.592)
Lag0–3	0.985 (0.900, 1.077)	0.995 (0.975, 1.015)	1.220 (1.032, 1.443)	1.271 (1.053, 1.534)
Lag0–4	1.006 (0.917, 1.104)	1.000 (0.979, 1.020)	1.184 (0.999, 1.404)	1.214 (1.002, 1.470)
Lag0–5	1.023 (0.931, 1.124)	1.003 (0.983, 1.024)	1.165 (0.988, 1.372)	1.180 (0.978, 1.422)
Lag0–6	1.028 (0.946, 1.117)	1.004 (0.986, 1.022)	1.179 (1.040, 1.336)	1.193 (1.030, 1.381)
Lag0–7	1.018 (0.942, 1.101)	1.001 (0.985, 1.018)	1.239 (1.145, 1.341)	1.268 (1.143, 1.407)

### 3.4 Subgroup analysis

**Figure 2:**
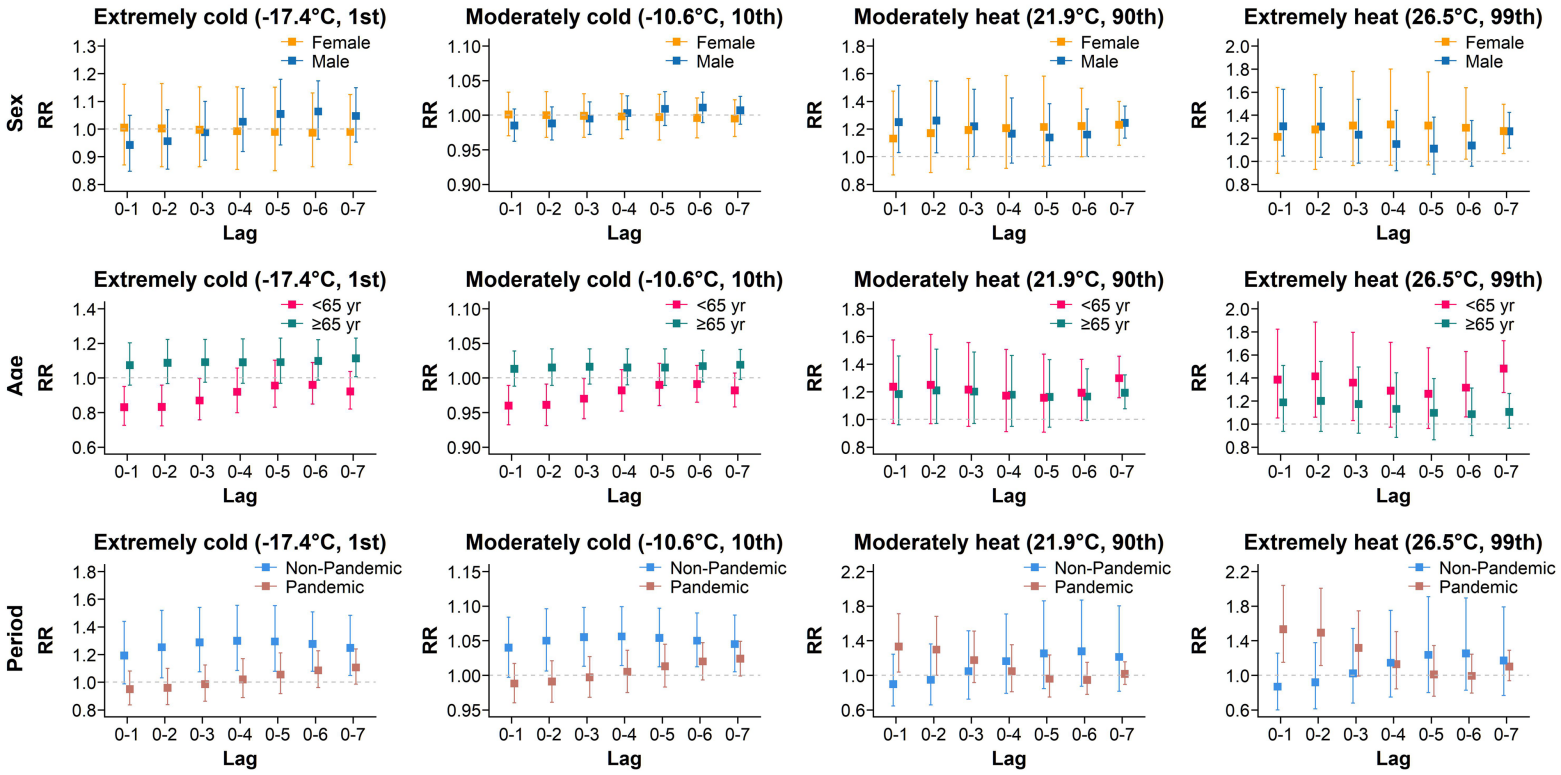
Cumulative effect of subgroup analysis within a lag of 0–7 days.

Considering that the time span of our study includes the COVID-19 pandemic, we conducted subgroup analyses based on sex (female, *N* = 5,355; male, *N* = 10,642), age (<65, *N* = 6,920; ≥65, *N* = 9,076; one patient whose age was missing in 2019), and the time period (non-pandemic, 2019 and 2023, *N* = 6,449; pandemic, 2020–2022, *N* = 9,548). The results are shown in Figure 2. In both extreme cold and moderate cold environments, no statistically significant increase in the AIS incidence was observed in males, females, or individuals <65 years old. Only after a lag of 7 days did extreme cold have adverse effects on individuals ≥65 years old. In contrast, in the early stages of lag, cold temperatures even have a protective effect against the development of AIS in individuals <65 years old. On day 2 of lag, the RR during the non-pandemic period was 1.252 (95% CI: 1.032–1.519), significantly higher than the RR of 0.959 (95% CI: 0.838–1.099) observed during the pandemic period. This disparity persisted on days 6 and 7 of lag.

Compared with cold temperatures, high temperatures were associated with a greater risk of developing AIS for both sex and age subgroups. Under extreme and moderate heat conditions, the impact of different lag periods on AIS risk displayed clear sex differences. Specifically, in the early lag period (2 days), an increased risk of AIS was observed among males. In contrast, females showed no significant change, suggesting that males may be more sensitive to short-term exposure to high temperatures. However, in the later lag stages (6 to 7 days), both sexes exhibited an elevated risk of AIS. As the lag period extended, the risk in females rose significantly, approaching or exceeding that in males. This finding suggests that females may be more sensitive to high temperatures over longer lag periods. Notably, moderately high temperatures had harmful effects on all age subgroups only after a lag of 7 days, with RRs of 1.298 (95% CI: 1.156–1.457) and 1.193 (95% CI: 1.077–1.321) for those <65 years old and those ≥65 years old, respectively. However, extreme heat had harmful effects on individuals <65 years old in the early stages of lag, reaching their maximum on the seventh lag day, with an RR of 1.480 (95% CI: 1.274–1.721). No heat-related harmful effects were detected among individuals ≥65 years old, indicating that exposure to high temperatures may have a greater impact on the risk of developing AIS in younger populations. Under high-temperature conditions, the highest risk of AIS occurrence during the pandemic period was observed on the first day of lag, with an RR of 1.331 (95% CI: 1.037–1.710) and 1.532 (95% CI: 1.150–2.040). In contrast, the harm caused by high temperature during the non-pandemic period was not significant.

### 3.5 Sensitivity analysis

We conducted several sensitivity analyses to assess the robustness of our findings. First, due to significant fluctuations in AIS case counts during the first half of 2019, we reanalyzed the data after excluding the period from January to June 2019. Second, we varied the degrees of freedom for the time variable (6, 8, 9) and for meteorological factors (5, 7) to test the stability of the model (Supplementary Figure S6). Third, we extended the maximum lag period to 14 days to capture potential longer-term lag effects (Supplementary Table S3). Finally, we used the mean daily minimum (1.4°C) and maximum (12.7°C) temperatures during the study period as alternative reference temperatures (Supplementary Table S4). The results showed no substantial changes in the estimated associations, suggesting that our findings are robust and reliable. The significant association between high temperatures and AIS incidence remained consistent across analyses.

## 4 Discussion

In the present study, we used the DLNM to explore the relationship between temperature and AIS incidence in Yanji, Northeast China. The results revealed that high temperature had a more immediate effect on the onset of AIS, with a significant lag effect. In the early stages of high-temperature exposure, males and patients <65 years old were more susceptible, and with prolonged lag times, high temperatures had harmful effects on all populations.

Temperature is widely believed to be associated with the onset and mortality of ischaemic stroke (28, 29). Research in Beijing, China, has shown that the cumulative effect of extreme cold (−10°C) on the onset of ischaemic stroke can be observed with lag and reaches its maximum on the fourteenth day, with an RR of 2.33 (95% CI: 1.16–4.92) (30). A cross-sectional case study conducted in Quebec, Canada, revealed that compared with those exposed to temperatures above 0°C, men exposed to −20°C had an approximate 1.2-fold greater risk of stroke the following day. Snowfall was also independently associated with the risk of haemorrhagic stroke death in men but not with ischaemic stroke (18). In this study, no statistically significant harmful effects of cold on the incidence of AIS in the entire population were observed. Possible explanations are as follows: First, previous studies have suggested that low temperatures are more likely to trigger haemorrhagic stroke, while high temperatures are primarily associated with an increased risk of ischaemic stroke (31–33). In the Beijing study, both ischaemic and haemorrhagic stroke cases were included; thus, the overall risk observed related to cold exposure may have been partially influenced by haemorrhagic stroke cases. Second, there are significant climatic differences between Beijing and Yanji. The minimum temperature in Beijing is −12°C, whereas in Yanji it can fall to −22.6°C. Due to prolonged exposure to colder environments, residents of Yanji may have developed greater physiological tolerance and adaptability to low temperatures. This adaptation may have mitigated the health risks associated with cold exposure while amplifying the adverse effects of high temperatures.

On the other hand, our findings show that AIS cases are predominantly concentrated between May and August each year. The overall exposure–lag–response curve of temperature and AIS incidence indicates that as the temperature increases, the AIS incidence significantly increases, suggesting a potential association between high temperature and the risk of developing AIS. Specifically, both moderate and extreme heat had harmful effects on the day of exposure, indicating that high temperatures not only have short-term and acute effects on the onset of AIS but also have sustained harmful effects that can be observed in the later stages of lag, suggesting that high temperatures may have a longer lag effect. Similar conclusions have also been reached in other regions. Research in Yancheng, China, has shown that the impact of extremely high temperatures (30.5°C) on hospitalization for AIS can be manifested on the day of exposure. Within lags of 0–3, 0–5, and 0–14 days, the RRs were 1.51 (95% CI: 1.31–1.75), 1.48 (95% CI: 1.26–1.73), and 1.27 (95% CI: 1.01–1.59) (20), respectively. Another national multicentre study in China revealed that the RR of AIS at a lag of 0–10 h was 1.88 (95% CI: 1.65–2.13) in extreme heat (33.3°C) (19). Research in South Korea also revealed that the increase in the number of ischaemic stroke cases is related to the daily maximum temperature but not to the daily minimum temperature (34).

Although high temperatures may increase the risk of developing AIS, there are certain differences in the cold and heat effects of studies in different regions, which may be related to factors such as study design, economic development level, chronic disease burden, personal health awareness, and geographical location. Of particular note among these factors is the geographical location (35). Research has shown that the RR of AIS in northern China is 1.80 (95% CI: 1.53–2.11), whereas that in southern China is 1.57 (95% CI: 1.31–1.87) (19). Research in South Korea has also revealed that the high-temperature effect is greater in high-latitude regions. For every 1°C increase, the risk of ischaemic stroke increases by 2–3% in southern cities and 4–5% in northern cities (32). Similar results have been observed in American cities (36). The speculated reason may be that residents in high-latitude regions have greater adaptability to cold environments, increasing their sensitivity to high temperatures. During the study period, the average temperature in Yanji, which is located in Northeast China, was only 6.9°C, which is lower than the average temperature in temperate and tropical regions. The adaptability of local residents to cold environments may increase their vulnerability to AIS in high-temperature environments.

The mechanism by which high temperatures lead to AIS remains unclear. It is hypothesized that exposure to high temperatures may elevate AIS risk through pathways such as haemoconcentration, thromboembolism, and inflammatory responses. Specifically, high-temperature conditions can lead to excessive sweating and significant loss of fluids and electrolytes, resulting in a water–electrolyte imbalance (37). This state may reduce blood volume, causing haemoconcentration, decreased blood flow velocity, and increased blood viscosity (38, 39), creating favourable conditions for thrombus formation. In addition, heat exposure may induce heat stress responses that activate the body’s inflammatory response system (40). These changes may further promote microvascular thrombosis and contribute to an increased risk of ischaemic stroke.

Sex-based subgroup analysis revealed that, compared with females, males with AIS are more susceptible to the effects of high temperatures in the early stages of exposure. This sex difference in temperature sensitivity may be attributed to physiological differences (41, 42), and over time, both males and females are affected by the harmful effects of high temperatures. In addition, the subgroup analysis of age revealed that high temperature had a greater effect on the incidence of AIS in people younger than 65 years, which may be related to the occupational exposure of young people, such as their participation in outdoor activities or physical labour. In addition, some studies have noted that the incidence of stroke among young people is increasing, and the number of early stroke cases is increasing (43). It appears that there are differences in the risk of AIS onset between the pandemic and non-pandemic periods. During periods of cold temperatures, the implementation of public health measures such as home quarantine and social distancing during the pandemic period significantly restricted people’s activity ranges. Consequently, the opportunities and duration of exposure to the cold outdoor environment were markedly reduced (44). In addition, the centralized indoor heating available in the northeastern region during winter may have further decreased the risk of AIS incidence triggered by cold weather. However, during the pandemic, the risk of AIS incidence under moderately high and extremely high temperatures was relatively higher in the initial period of exposure. The public health measures that encouraged people to stay indoors led to prolonged exposure to indoor environments. Under high temperatures, indoor temperatures could be higher, especially in households lacking effective cooling devices. Prolonged exposure to high indoor temperatures increased the likelihood of AIS incidence. Moreover, the psychological stress during the pandemic could be exacerbated in high temperatures. The combination of psychological and physiological stress was particularly pronounced in high temperatures, further exacerbating the risks.

Our findings may support the development of early warning systems for temperature-related health risks, such as issuing alerts during extreme heat events to enable timely public health responses. In addition, health advisories can be provided to high-risk populations—such as the older adult or individuals with pre-existing cardiovascular conditions—to encourage protective behaviors (e.g., avoiding outdoor activities during hot periods, staying well-hydrated, or seeking cooler environments). These insights may also inform the design of region-specific, temperature-sensitive stroke prevention strategies, ultimately contributing to a reduction in AIS incidence and alleviating the burden on healthcare systems.

Our study has several limitations. First, the retrospective design restricts our ability to establish a causal relationship between temperature and the incidence of AIS. Second, due to limited data sources, we only collected data from a single hospital in Yanji, and information on potential confounding factors beyond age and sex—such as health-related behaviors and comorbidities—was not available. Third, the study as a whole is an ecological study that can only be observed at the population level and cannot obtain accurate individual exposure values, which introduces the risk of ecological fallacy. Moreover, previous studies have shown that environmental pollutants (e.g., PM_2.5_ and PM_10_) may also be associated with AIS incidence; however, these factors were not considered in our analysis. Finally, because of differences in study design and the variables included in the model, caution is warranted when generalizing our findings to other regions.

## 5 Conclusion

Our research results indicate a nonlinear correlation between temperature and the incidence of AIS. High temperature increases the risk of developing AIS. In the early stages of exposure to high temperature, the risk of developing AIS in males and those <65 years old increases, and as the lag time increases, high temperature has adverse effects on the entire population. These findings provide an important basis for the development of AIS prevention and control strategies in Yanji under different weather conditions, and reducing the exposure of the population to high temperature may alleviate the medical burden caused by AIS.

## Data Availability

The datasets presented in this article are not readily available because considering ethical standards, the data is specific to this study only. Requests to access the datasets should be directed to Binyu Zhao, zhaobinyu062@163.com.
