# Supplementary material for: The correlation between temperature and the incidence of acute ischaemic stroke in Yanji, China: a time series study
Source: Front Public Health. 2025 Jul 29;13:1568759. doi: 10.3389/fpubh.2025.1568759 (PMC12339521; doi:10.3389/fpubh.2025.1568759)
Supplement: Supplementary file 1 [file Data_Sheet_1.docx]

Supplementary Material

Contents

[Figure S1. Geographic location of Yanji City in the People's Republic of China 2](#_Toc17866)

[Table S1. Correlation tests among meteorological factors 3](#_Toc3283)

[Table S2. Multicollinearity tests among meteorological factors 3](#_Toc20314)

[Figure S2. Model residual normality test Q-Q plot. 4](#_Toc26000)

[Figure S3. The spline function for estimating the minimum morbidity temperature (MMT) 5](#_Toc23418)

[Figure S4. Monthly AIS cases during the study period. 6](#_Toc6726)

[Figure S5. Series plots of the meteorological variables during the study period. 7](#_Toc23990)

[Figure S6. The results of sensitivity analysis by changing degrees of freedom 8](#_Toc21047)

[Table S3. The results of sensitivity analysis when the maximum lag period was increased to 14 days 9](#_Toc6831)

[Table S4. The results of sensitivity analysis using mean daily minimum (1.4 °C) and maximum (12.7 °C) temperatures as reference temperature 10](#_Toc1240)

[References 11](#_Toc15963)


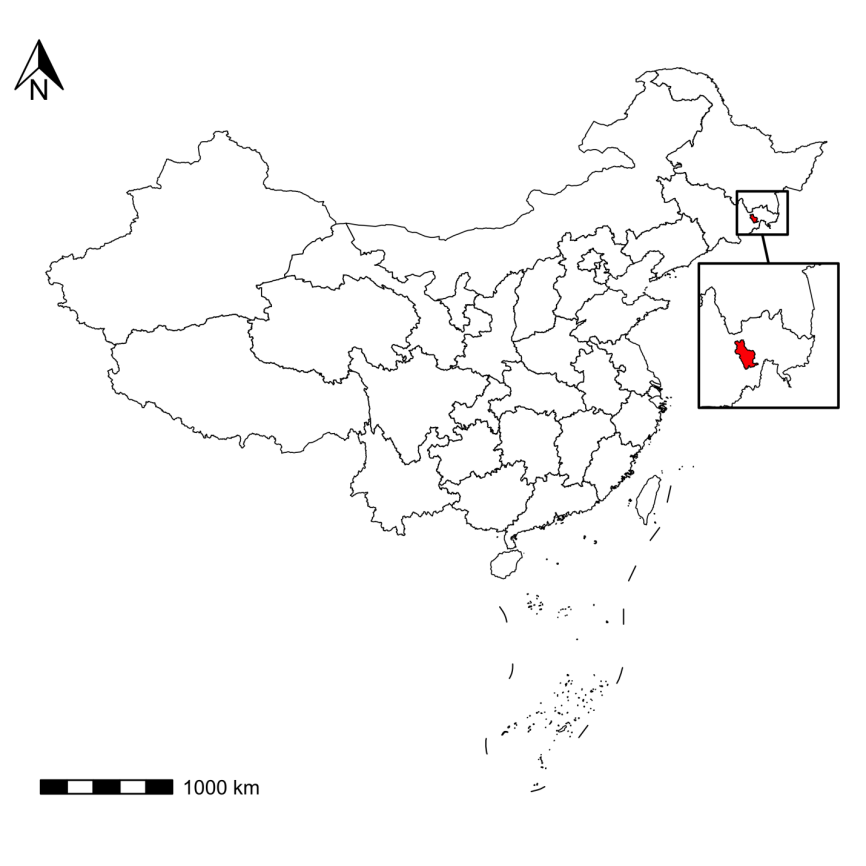


**Figure S1.** Geographic location of Yanji City in the People's Republic of China

**Table S1.** Correlation tests among meteorological factors

|  | Temperature | Relative humidity | Precipitation | Wind speed | Air pressure | AIS cases |
| --- | --- | --- | --- | --- | --- | --- |
| Temperature | 1.000 |  |  |  |  |  |
| Relative humidity | 0.550^**^ | 1.000 |  |  |  |  |
| Precipitation | 0.394^**^ | 0.617^**^ | 1.000 |  |  |  |
| Wind speed | -0.231^**^ | -0.350^**^ | -0.032 | 1.000 |  |  |
| Air pressure | -0.745^**^ | -0.454^**^ | -0.535^**^ | -0.051^*^ | 1.000 |  |
| AIS cases | 0.209^**^ | 0.213^**^ | 0.110^**^ | -0.126^**^ | -0.133^**^ | 1.000 |

^*^*P*-value < 0.05; ^**^*P*-value < 0.001.

**Table S2.** Multicollinearity tests among meteorological factors

|  | VIF^a^ | GVIF^(1/(2*Df))^b^ |
| --- | --- | --- |
| Temperature^a^ / *T_t,l_^b^* | 2.733591 | 1.070982 |
| Relative humidity | 1.748233 | 1.419120 |
| Precipitation | 1.234652 | 1.116637 |
| Wind speed | 1.386678 | 1.174633 |
| Air pressure | 2.775439 | 1.664016 |

Abbreviations: VIF = Variance Inflation Factor; GVIF = Generalized Variance Inflation Factor.

In Table S2. VIF should be less than 5, GVIF should be less than 2, which is a conservative threshhold for considering multicollinearity^1^.


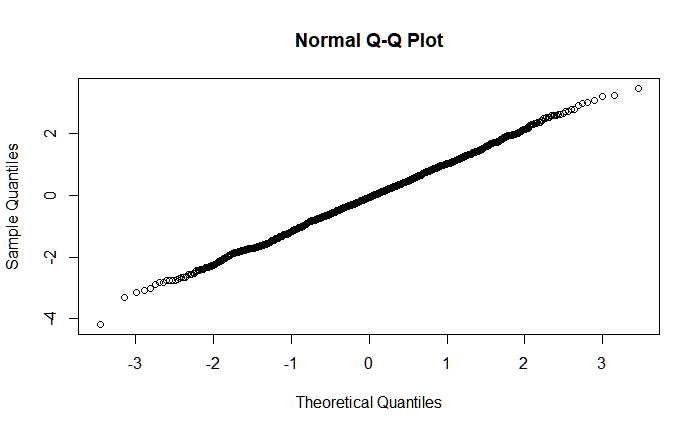


**Figure S2.** Model residual normality test Q-Q plot.

The Shapiro-Wilk normality test results are as follows:

Shapiro-Wilk normality test

data: residuals

W = 0.99934, p-value = 0.8043

1. "Shapiro-Wilk test p-value: 0.804349942334799"


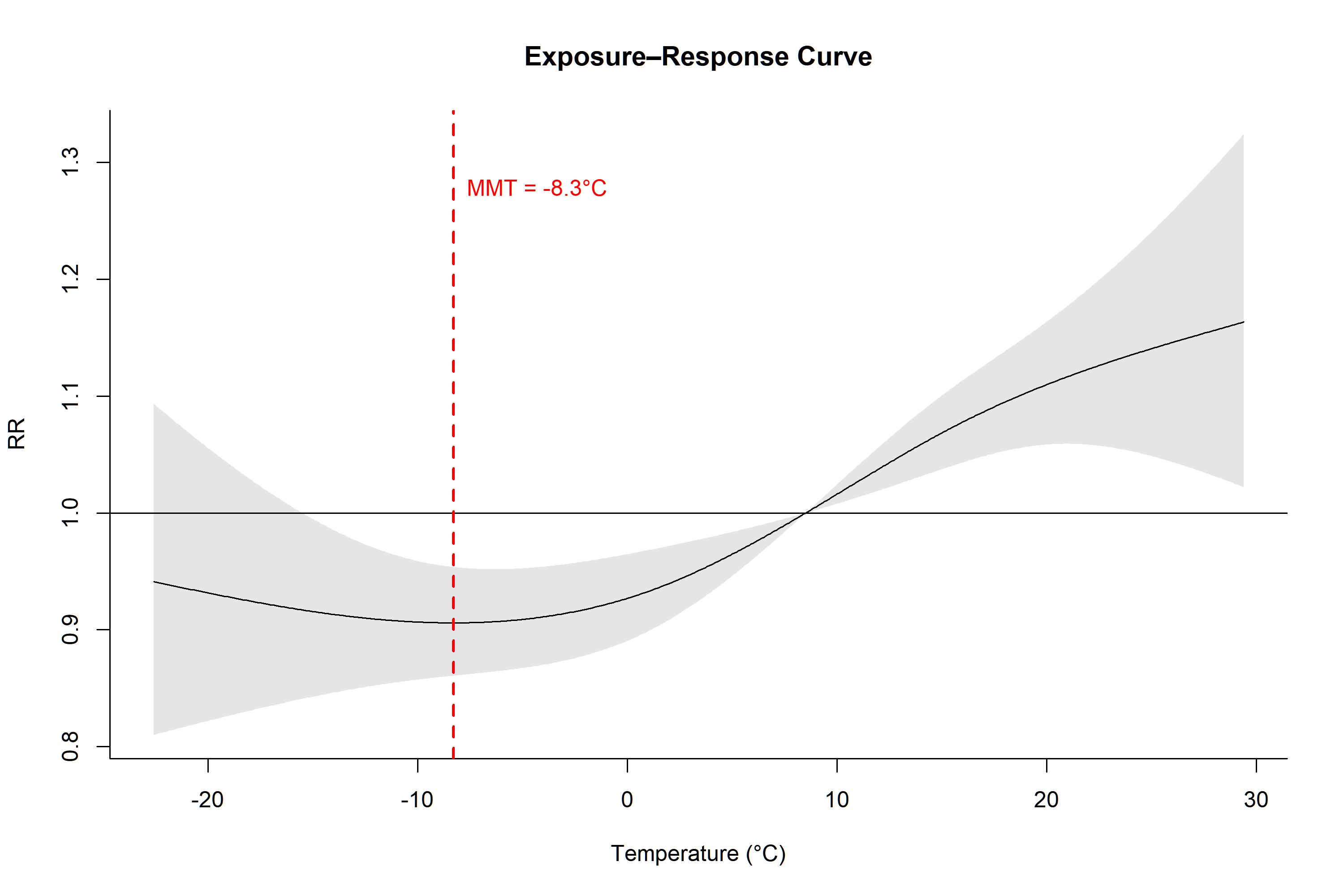


**Figure S3.** The spline function for estimating the minimum morbidity temperature (MMT)

The R code for estimating the Minimum Morbidity Temperature (MMT) is available in the supplementary materials of “*Tobías A, Armstrong B, and Gasparrini A. Brief Report: Investigating Uncertainty in the Minimum Mortality Temperature: Methods and Application to 52 Spanish Cities. Epidemiology (2017) 28(1):72-6. Epub 2016/10/18. doi: 10.1097/ede.0000000000000567*”.

**
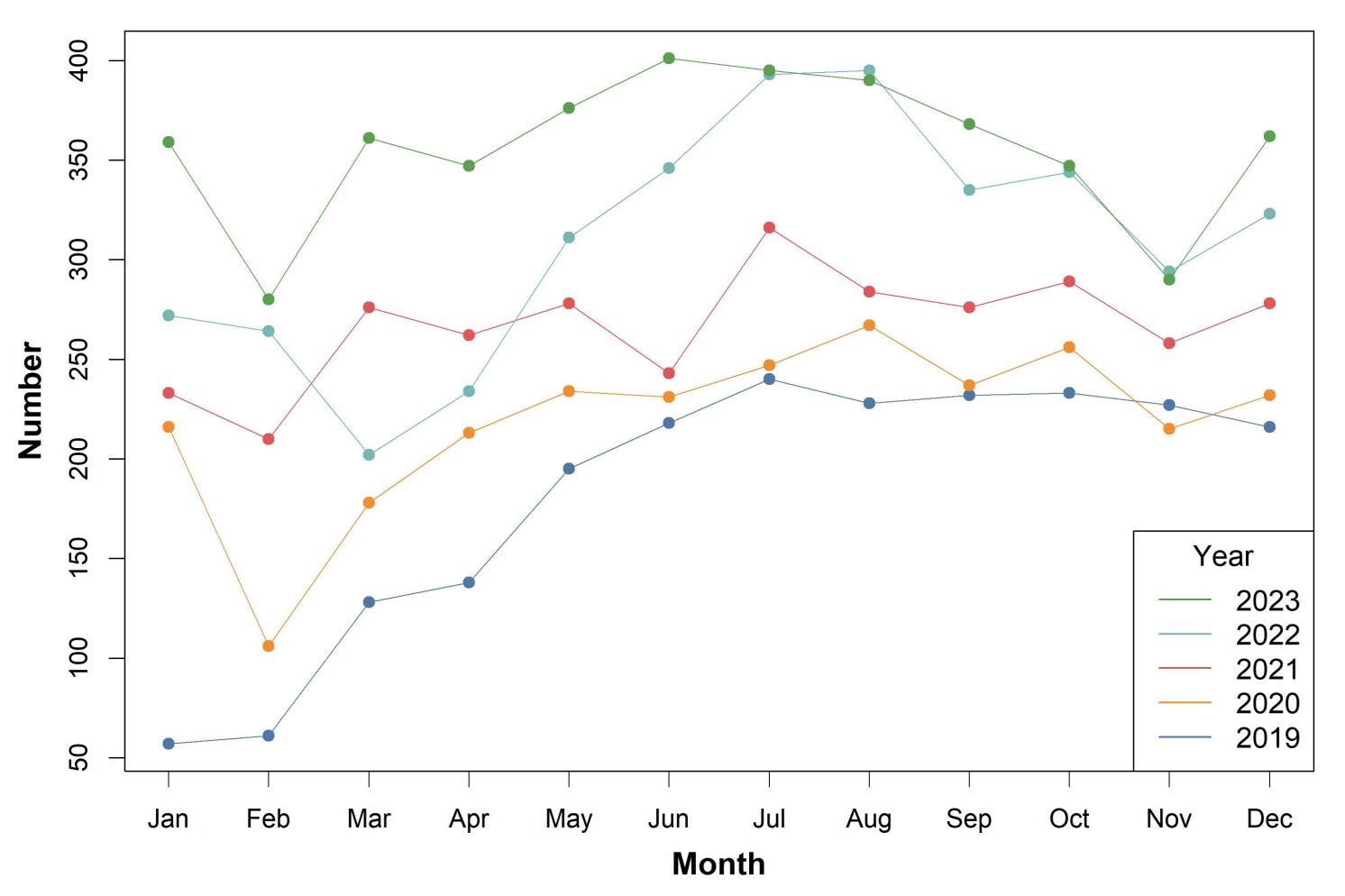
**

**Figure S4.** Monthly AIS cases during the study period.

**
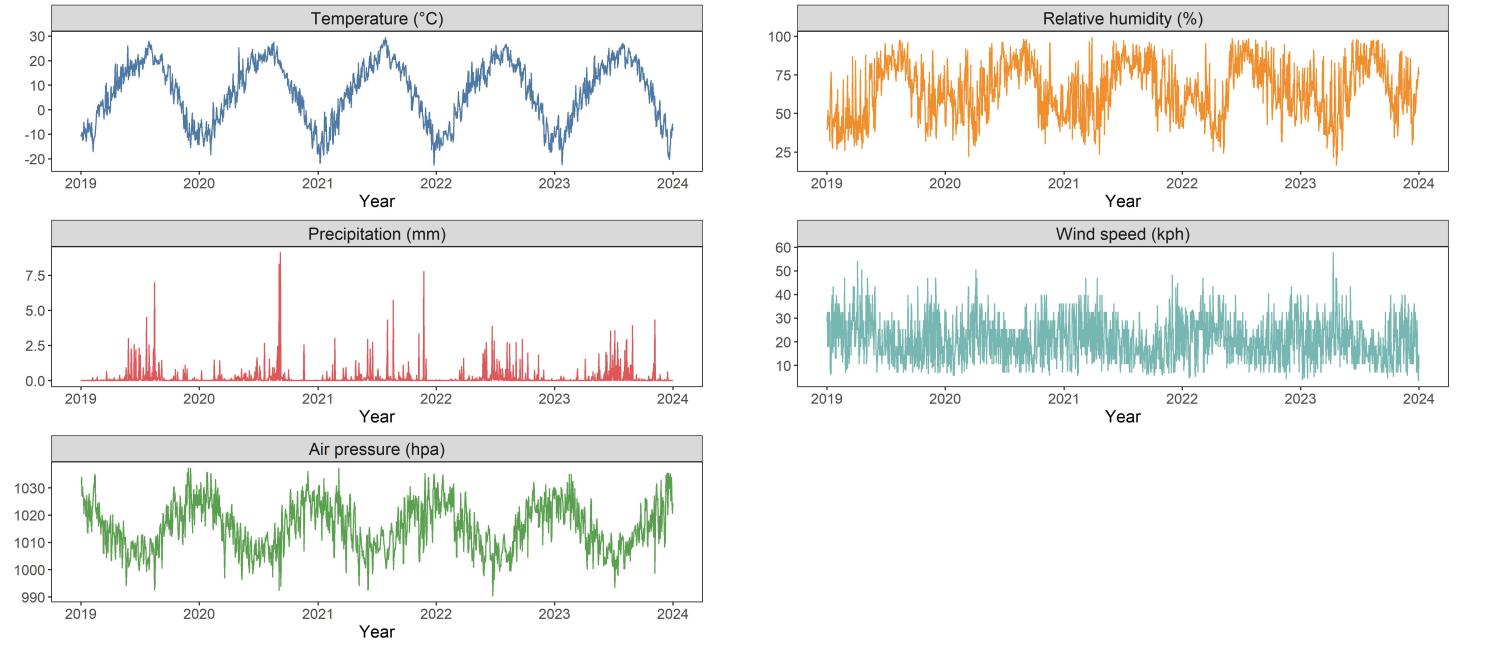
**

**Figure S5.** Series plots of the meteorological variables during the study period.

**
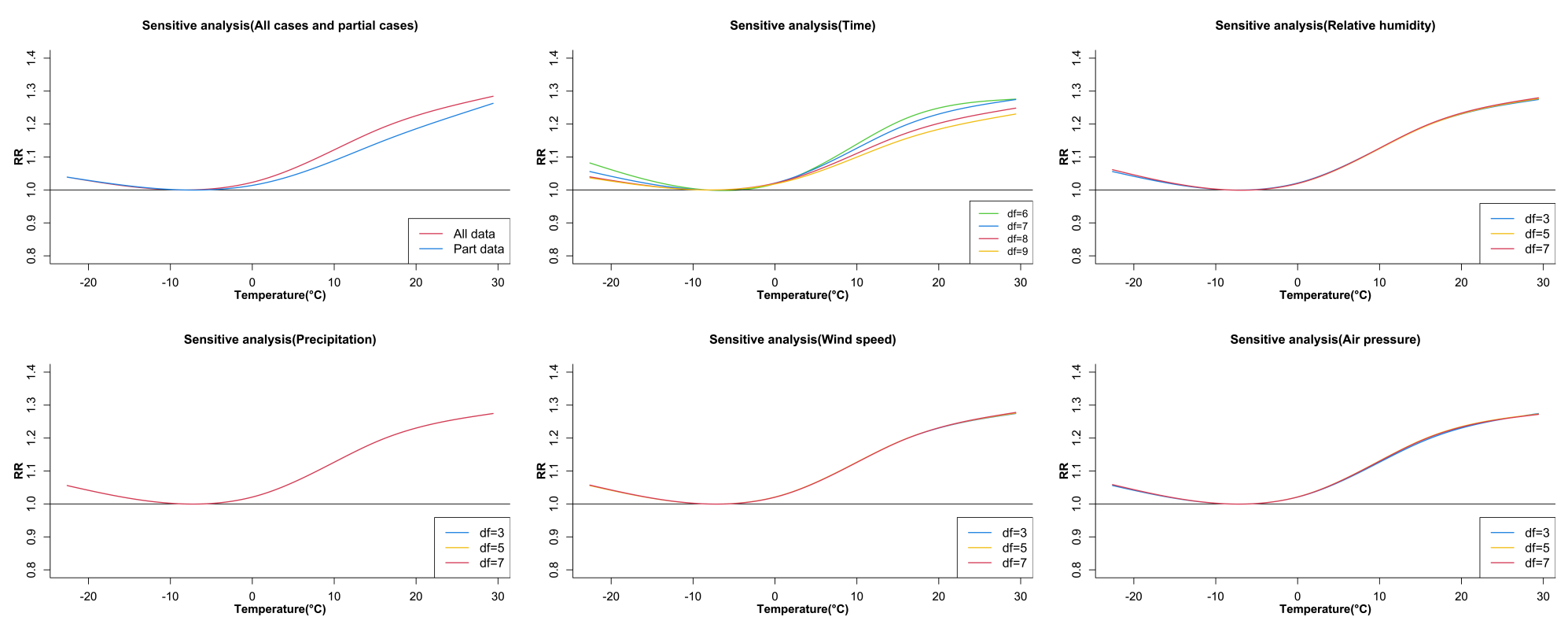
**

**Figure S6.** The results of sensitivity analysis by changing degrees of freedom

**Table S3.** The results of sensitivity analysis when the maximum lag period was increased to 14 days

| **Lag** | **Extremely cold**  **-17.4 °C, 1st** | **Moderately cold -10.6 °C, 10th** | **Moderately hot**  **21.9 °C, 90th** | **Extremely hot 26.5 °C, 99th** |
| --- | --- | --- | --- | --- |
| Lag0-0 | 0.984 (0.947, 1.023) | 0.995 (0.984, 1.006) | 1.045 (0.977, 1.119) | 1.060 (0.983, 1.144) |
| Lag0-1 | 0.976 (0.914, 1.043) | 0.992 (0.973, 1.012) | 1.083 (0.963, 1.217) | 1.112 (0.976, 1.267) |
| Lag0-2 | 0.976 (0.897, 1.062) | 0.992 (0.968, 1.017) | 1.112 (0.957, 1.291) | 1.153 (0.977, 1.362) |
| Lag0-3 | 0.982 (0.894, 1.079) | 0.994 (0.967, 1.022) | 1.132 (0.957, 1.338) | 1.184 (0.984, 1.426) |
| Lag0-4 | 0.995 (0.902, 1.096) | 0.997 (0.969, 1.026) | 1.144 (0.960, 1.362) | 1.205 (0.994, 1.461) |
| Lag0-5 | 1.011 (0.918, 1.114) | 1.002 (0.974, 1.031) | 1.149 (0.964, 1.369) | **1.216 (1.004, 1.474)** |
| Lag0-6 | 1.031 (0.936, 1.135) | 1.008 (0.980, 1.037) | 1.149 (0.966, 1.367) | **1.220 (1.009, 1.474)** |
| Lag0-7 | 1.051 (0.954, 1.158) | 1.014 (0.985, 1.043) | 1.147 (0.964, 1.363) | **1.218 (1.008, 1.472)** |
| Lag0-8 | 1.069 (0.968, 1.181) | 1.019 (0.990, 1.049) | 1.144 (0.961, 1.362) | **1.213 (1.001, 1.469)** |
| Lag0-9 | 1.083 (0.978, 1.200) | 1.022 (0.993, 1.053) | 1.143 (0.960, 1.361) | 1.208 (0.995, 1.465) |
| Lag0-10 | 1.091 (0.984, 1.209) | 1.024 (0.994, 1.055) | 1.146 (0.967, 1.358) | 1.204 (0.996, 1.455) |
| Lag0-11 | 1.090 (0.987, 1.205) | 1.024 (0.994, 1.054) | 1.154 (0.988, 1.348) | **1.203 (1.009, 1.434)** |
| Lag0-12 | 1.080 (0.983, 1.187) | 1.020 (0.993, 1.048) | **1.169 (1.026, 1.332)** | **1.206 (1.037, 1.402)** |
| Lag0-13 | 1.060 (0.969, 1.160) | 1.014 (0.988, 1.040) | **1.192 (1.081, 1.314)** | **1.214 (1.077, 1.367)** |
| Lag0-14 | 1.031 (0.937, 1.133) | 1.004 (0.978, 1.031) | **1.223 (1.131, 1.322)** | **1.227 (1.105, 1.363)** |

**Table S4.** The results of sensitivity analysis using mean daily minimum (1.4 °C) and maximum (12.7 °C) temperatures as reference temperature

| **Lag** | **Extremely cold**  **-17.4 °C, 1st** | **Moderately cold -10.6 °C, 10th** | **Moderately hot**  **21.9 °C, 90th** | **Extremely hot 26.5 °C, 99th** |
| --- | --- | --- | --- | --- |
| **Ref=1.4 °C** |  |  |  |  |
| Lag0-0 | 0.940 (0.852, 1.037) | 0.962 (0.911, 1.016) | **1.104 (1.011, 1.206)** | **1.146 (1.027, 1.279)** |
| Lag0-1 | 0.916 (0.794, 1.057) | 0.945 (0.873, 1.023) | **1.166 (1.025, 1.327)** | **1.231 (1.050, 1.444)** |
| Lag0-2 | 0.922 (0.795, 1.070) | 0.945 (0.871, 1.027) | **1.186 (1.035, 1.359)** | **1.251 (1.059, 1.477)** |
| Lag0-3 | 0.948 (0.822, 1.092) | 0.957 (0.885, 1.036) | **1.175 (1.028, 1.341)** | **1.223 (1.041, 1.437)** |
| Lag0-4 | 0.980 (0.847, 1.133) | 0.973 (0.898, 1.054) | **1.153 (1.006, 1.321)** | **1.182 (1.001, 1.395)** |
| Lag0-5 | 1.003 (0.867, 1.160) | 0.984 (0.908, 1.065) | 1.142 (1.000, 1.304) | 1.157 (0.982, 1.363) |
| Lag0-6 | 1.007 (0.889, 1.141) | 0.984 (0.922, 1.050) | **1.155 (1.041, 1.282)** | **1.168 (1.025, 1.332)** |
| Lag0-7 | 0.986 (0.883, 1.102) | 0.970 (0.921, 1.022) | **1.200 (1.115, 1.292)** | **1.228 (1.112, 1.355)** |
| **Ref=12.7 °C** |  |  |  |  |
| Lag0-0 | 0.902 (0.798, 1.020) | 0.923 (0.838, 1.018) | **1.060 (1.008, 1.115)** | **1.100 (1.005, 1.204)** |
| Lag0-1 | 0.858 (0.718, 1.025) | 0.884 (0.767, 1.019) | **1.092 (1.014, 1.175)** | **1.152 (1.011, 1.313)** |
| Lag0-2 | 0.852 (0.708, 1.026) | 0.874 (0.753, 1.015) | **1.096 (1.016, 1.183)** | **1.156 (1.010, 1.323)** |
| Lag0-3 | 0.873 (0.730, 1.044) | 0.882 (0.763, 1.021) | **1.082 (1.006, 1.165)** | 1.127 (0.989, 1.285) |
| Lag0-4 | 0.903 (0.753, 1.083) | 0.897 (0.773, 1.040) | 1.063 (0.985, 1.147) | 1.089 (0.950, 1.249) |
| Lag0-5 | 0.923 (0.773, 1.101) | 0.905 (0.784, 1.044) | 1.050 (0.973, 1.134) | 1.064 (0.926, 1.222) |
| Lag0-6 | 0.918 (0.799, 1.055) | **0.896 (0.804, 0.999)** | 1.053 (0.988, 1.121) | 1.065 (0.948, 1.196) |
| Lag0-7 | **0.883 (0.800, 0.973)** | **0.868 (0.812, 0.928)** | **1.074 (1.020, 1.131)** | 1.099 (0.997, 1.211) |

**References**

1. Fox J, Monette G. Generalized collinearity diagnostics. *J Am Stat Assoc*. 1992;87:178–183. doi: 10.1080/01621459.1992.10475190
